# The lymphatic vascular system of the mouse head

**DOI:** 10.1007/s00441-016-2493-8

**Published:** 2016-09-06

**Authors:** Melanie Lohrberg, Jörg Wilting

**Affiliations:** Department of Anatomy and Cell Biology, University Medical School Göttingen, Göttingen, Germany

**Keywords:** Lymphatic endothelial cells, Conjunctiva, Oral mucous membrane, Dura mater, Nasolacrimal duct

## Abstract

Histological studies of the lymphatic vascular system in adult mice are hampered because bones cannot be sectioned properly. Here, we decalcified the heads of 14-day-old mice, embedded them in paraffin and stained resultant serial sections with the lymphendothelial-specific antibodies Lyve-1 and Podoplanin. We show that the tissues with the highest lymphatic vascular density are the dermis and the oral mucous membranes. In contrast, the nasal mucous membrane is devoid of lymphatics, except for its most basal parts below the vomeronasal organ. The inferior nasal turbinate contains numerous lymphatics and is connected to the nasolacrimal duct (NLD), which is ensheathed by a dense network of lymphatics. The lymphatics of the eye lids and conjunctiva are connected to those of the inferior nasal turbinate. We suggest that cerebro-spinal fluid (CSF) can drain via the optic nerve and NLD lymphatics, whereas CSF drained via the *Fila olfactoria* into the nasal mucous membrane is used for moisturization of the respiratory air. Tongue, palatine and buccal mucous membranes possess numerous lymphatics, whereas the dental pulp has none. Lymphatics are present in the maxillary gland and close to the temporomandibular joint, suggesting the augmentation of lymph flow by chewing and yawning. Lymphatics can also be found in the dura mater and in the dural septae entering into deeper parts of the brain. Our findings are discussed with regard to CSF drainage and potential routes for ocular tumor dissemination.

## Introduction

Because of its functions in immune surveillance, the lymphatic vascular system has received increasing interest in recent years (Tammela and Alitalo 2010). An insufficiency of the lymphatic vascular network causes lymphedema and reduces the circulation of leukocytes, which prepares the ground for infections such as painful group-A streptococcal infections of the skin (erysipelas; Witte et al. 2001). Immune surveillance of the central nervous system (CNS) appears to be different from that of most other organs as it is an immuno-privileged organ protected by the blood–brain-barrier (BBB) and controlled by microglia and endogenously re-circulating T-lymphocytes (Goldmann 1913; Schläger et al. 2016). However, during ageing the integrity of the BBB becomes reduced (Zeevi et al. 2010). The lymphatic drainage of the CNS has been studied for decades. Markers injected into the cerebrospinal fluid (CSF) of the brain are detectable within minutes in the deep cervical lymph nodes (Abbott 2004; Cserr et al. 1992), indicating that the cranial and cervical nerves serve as the main drainage pathways (Johnston et al. 2004; Lüdemann et al. 2005). The first to claim an affiliation between CSF and the lymphovascular system were the German anatomists Wilhelm His and Gustav Schwalbe, who performed injections of Berlin-blue into the CNS of dogs (His 1865; Schwalbe 1869). Their results have since been confirmed in many other species. However, the pattern of lymphatics in the head, taking up and conducting CSF and other interstitial fluids, is still not completely understood.

Recently, a dural lymphatic vascular system has been published questioning the prevailing dogma that the CNS is, besides the placenta, the only organ system without a lymphovascular system (Aspelund et al. 2015; Louveau et al. 2015). These studies have been performed in wild-type mice and in transgenic mice carrying lymphatic endothelial cell (LEC)-specific reporters and have provided evidence for a functional lymphovascular system in the dura mater. The two investigations involved the use of the whole-mount staining of the lymphatics of dissected murine heads, a method that provides an excellent spatial overview but that does not provide histological cellular resolution. We therefore sectioned complete heads of mice after decalcification of the bony structures and stained the resultant sections with LEC-specific antibodies that still work after this pretreatment. In this study, we describe the pattern of lymphatics in the murine head.

## Materials and methods

### Animals

For these studies, we used 14-day-old NMRI mice (*n* = 12). All experiments were reported to our local institutional animal care committee and the Lower Saxony State Council on Animal Care (LAVES). The studies corresponded to the requirements of the American Physiological Society.

### Tissue preparation

The 14-day-old mice were sacrificed and their heads were dissected accordingly and further prepared for paraffin- or cryo-embedding. For paraffin-embedding, heads were fixed for 24 h in 4 % paraformaldehyde (PFA). The fixed heads were then decalcified for 14 days in 1 M ethylenediamine tetraacetic acid (EDTA) solution. The EDTA solution was changed every other day. Heads were embedded in paraffin and 5-μm-thick coronal slices were cut for immunohistochemistry (*n* = 4). For cryo-embedding, the skull was opened and the brain was removed and briefly fixed in 4 % PFA for 25 min. The brains were subsequently embedded in Polyfreeze Tissue Freezing Medium (Polysciences) and stored at −20 °C. By means of a cryostat (Leica CM 3050S), 16-μm-thick coronal slides were cut for immunofluorescence and stored at −20 °C (*n* = 8).

### Immunohistochemistry

Sections were deparaffinized in xylene and rehydrated in alcohol solutions with descending concentrations. Afterwards, the slices were rinsed in phosphate-buffered saline (PBS) and endogenous peroxidase was quenched by incubation for 30 min with 0.3 % H_2_O_2_ in methanol at room temperature. To prevent non-specific binding of antibodies, sections were incubated with 1 % bovine serum albumin (20 min, room temperature; AppliChem). The primary antibodies (rabbit-anti-mouse, anti-Lyve-1, Lot 1210R03, ReliaTech, Wolfenbüttel, Germany; or hamster-anti-mouse, anti-Podoplanin, RTD4E10, abcam), diluted 1:300–1:800 in blocking buffer, were added and the slides were incubated overnight at 4 °C. In negative controls, the primary antibody was omitted. On the following day, the slides were washed in PBS and afterwards, a peroxidase-conjugated goat-anti-mouse secondary antibody (Lot 098 K6035, Sigma) diluted 1:200 (v/v) in PBS or a peroxidase-conjugated goat-anti-hamster secondary antibody (abcam) diluted 1:500 in PBS was added to the sections, which were then incubated for 20 min at room temperature. Then, the sections were rinsed with PBS and stained with 3,3’-diaminobenzidine (DAB, Sigma) for 8 min. Finally, the sections were counterstained for 20 s in nuclear fast red (Merck), mounted and evaluated by using a light microscope (Olympus CX21) equipped with a digital imaging system (Olympus U-CMAD3 camera, IC Capture 2.2). Pictures were processed by using AdobePhotoshop 2.2.

### Immunofluorescence

Sections were dried for 1 h at room temperature and afterwards, non-specific binding sites were blocked by incubation with 1 % bovine serum albumin (20 min, room temperature; AppliChem). The primary antibodies, namely rabbit polyclonal anti-human-Prox-1 (Lot 0810R19-1, ReliaTech), purified rat anti-mouse-CD31 (clone MEC13.3, BD Pharmingen), rabbit-anti-mouse-Lyve-1 (Lot 1210R03, ReliaTech), or hamster-anti-mouse-Podoplanin (RTD4E10, abcam), diluted 1:500 (anti-Prox-1, anti-Lyve-1, and anti-Podoplanin) or 1:50 (anti-CD31) in blocking buffer, were added. The slides were incubated overnight at 4 °C. In negative controls, the primary antibody was omitted. On the following day, the slides were washed in PBS and afterwards, fluorescence-labeled secondary antibodies (goat anti-rat Alexa488 IgG [H + L], Invitrogen; goat anti-rabbit Alexa594 IgG [H + L], Invitrogen, or goat anti-hamster Alexa594 IgG [H + L], Invitrogen), diluted 1:200 (v/v) in blocking buffer, were added. For nuclear staining, 4,6-diamidino-2-phenylindole (Dapi; Invitrogen) was added at a dilution of 1:10,000. Slides were incubated for 1 h at room temperature. Then, the sections were rinsed with PBS, mounted in Fluoromount-G (SouthernBiotech) and evaluated by using a fluorescence microscope (AxioImager.Z1, Zeiss) equipped with a digital imaging system (AxioCam MRm and AxioVision Software, Zeiss). Pictures were processed by using AdobePhotoshop 2.2.

## Results

The tissues with the highest lymphatic vascular density in the mouse head are known to be the skin and the oral mucous membranes. The dermis contains a dense network of initial lymphatics that nicely depicts the specificity of the used anti-Lyve-1 antibody (data not shown). The dermis of the snout also contains numerous lymphatics; however, the whiskers are separated from the lymphovascular system by a capsule of dense connective tissue. Drainage of the lymph from the dermis obviously takes place by lymphatic collectors localized adjacent to superficial arteries, such as the facial artery and the superficial temporal artery (data not shown).

**Fig. 1:**
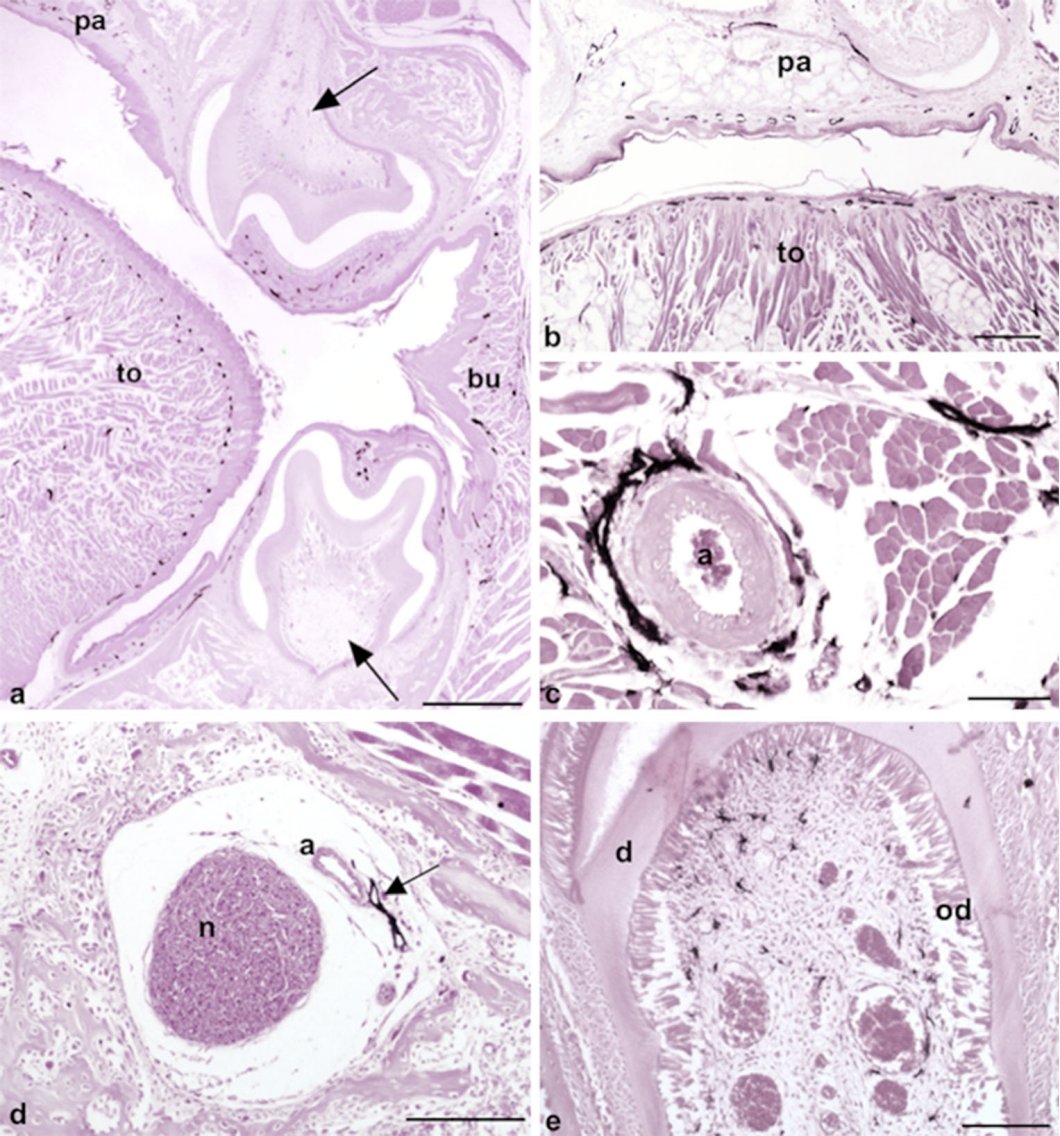
Oral mucous membrane containing a dense network of initial lymphatics. **a** Initial lymphatics are localized in a subepithelial position in the tongue (*to*), gingiva, cheek with buccinator muscle (*bu*) and palatine (*pa*). Dental pulp (*arrows*) does not contain lymphatics. Magnification ×20. *Bar* 400 μm. **b** Root of the tongue (*to*) and palatine (*pa*) with a subepithelial lymphatic network. Magnification ×40. *Bar* 200 μm. **c** Lymphatics along the lingual artery (*a*). Magnification ×200. *Bar* 50 μm. **d** Lymphatic vessel (*arrow*) accompanying the inferior alveolar artery (*a*) and nerve (*n*). Magnification ×100. *Bar* 100 μm. **e** Dendritic cells in dental pulp are weakly positive for Lyve-1 (*d* dentin, *od* odontoblasts). Magnification ×100. *Bar* 100 μm

**Fig. 2:**
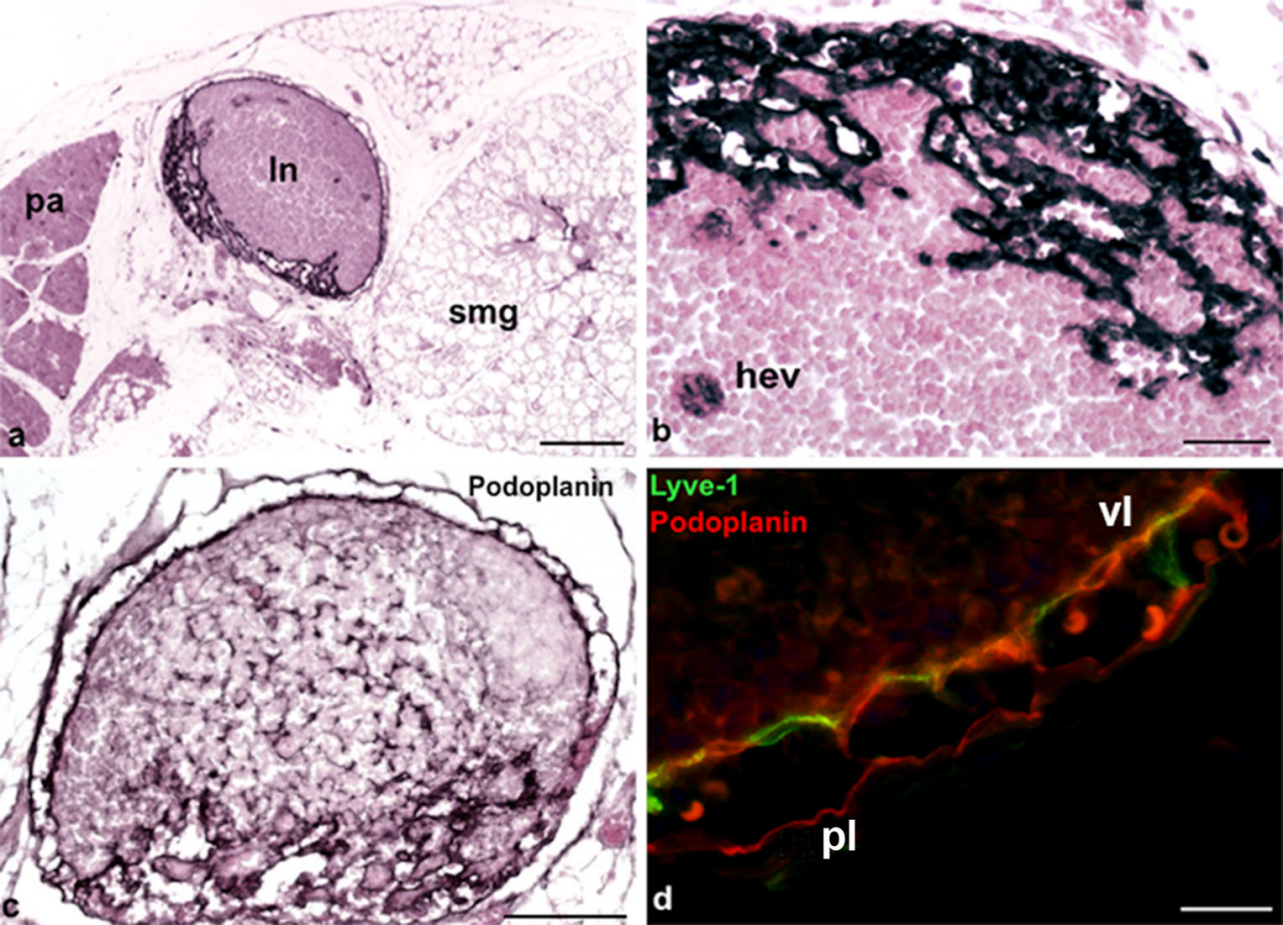
Lyve-1 expression in dendritic cells, high endothelial venules (HEVs) and LECs. **a** Lymph node (*ln*) located between the parotis (*pa*) and the submandibular gland (*smg*). Magnification ×40. *Bar* 200 μm. **b** Lymph node showing Lyve-1 expression in HEVs (*hev*). Magnification ×200. *Bar* 50 μm. **c** Anti-podoplanin staining of the same lymph node as that in **b**. Note the podopanin^+^ reticular cells and podoplanin^+^ sinuses. Magnification ×100. *Bar* 100 μm. **d** Immunofluorescence staining of a lymph node with the antibodies anti-Lyve-1 (*green*) and anti-podoplanin (*red*). Note the Lyve-1^+^/Podoplanin^+^ visceral layer (*vl*) and the Lyve-1^−^/Podoplanin^+^ parietal layer (*pl*). Magnification ×400. *Bar* 20 μm

As previously indicated, the oral mucous membrane possesses a dense network of initial lymphatics that can be found in all its regions including its gingival, buccal and palatinal aspects (Fig. 1a,b). In the tongue, these lymphatics are situated immediately beneath the epithelial layer, both at the back of the tongue and in its floor. Additionally, the tongue muscle contains numerous lymphatics that most likely drain into collectors accompanying the lingual artery (Fig. 1a-c). Interestingly, the dental pulp is free of lymphatics (Fig. 1a) but lymphatics can be found accompanying the inferior alveolar artery and nerve (Fig. 1d). Although we did not find any lymphatics in the dental pulp, we observed ramified cells weakly positive for Lyve-1, which might have represented dendritic cells or macrophages (Fig. 1e). At the floor of the mouth, lymph nodes are found in between the submandibular and parotid glands (Fig. 2a). These murine lymph nodes possess a marginal sinus. However, although both layers were Podoplanin-positive (Fig. 2c), only the visceral layer of the sinus was Lyve-1-positive, whereas the parietal layer was mostly Lyve-1-negative (Fig. 2d). Trabecular sinuses, as found in human lymph nodes, are not present. In the center of the lymph nodes, high endothelial venules (HEVs) are found to be weakly positive for Lyve-1 (Fig. 2b). Reticular cells are detectable as being Podoplanin-positive (Fig. 2c). We found more lymphatics in the submandibular gland as compared with the parotis and these lymphatics were usually located adjacent to the intra- and interlobular excretory ducts (data not shown). Lymphatics were also located in close proximity to the temporomandibular joint, suggesting a function for the uptake of synovial fluid. Contrarily, the number of lymphatics in the masseteric muscle was very low (data not shown).

**Fig. 3:**
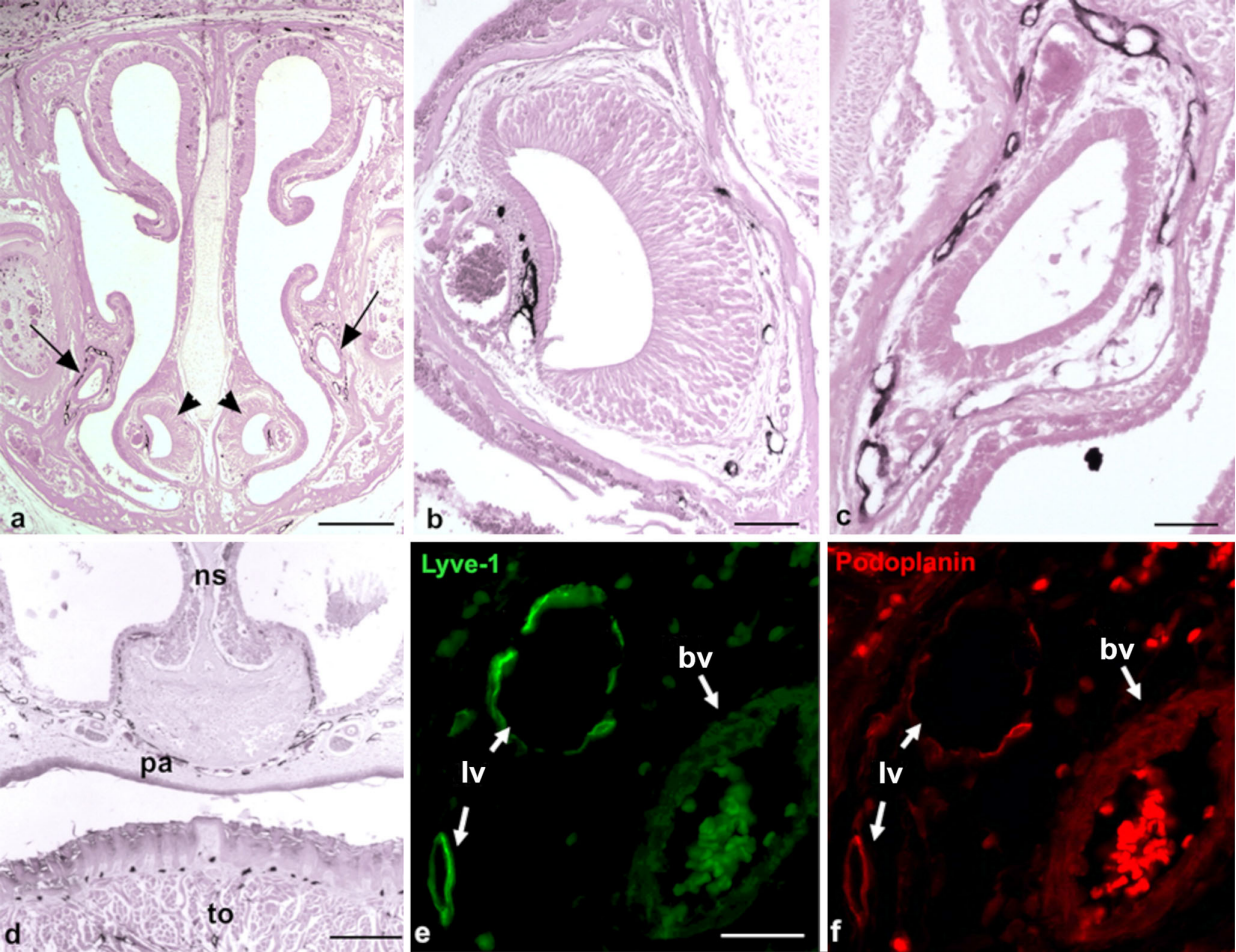
Lyve-1-positive lymphatics in the basal parts of the nasal mucous membrane and along the nasolacrimal duct. **a** Overview of the nasal mucous membrane showing the position of the vomeronasal organ (*arrowheads*) and the nasolacrimal duct (*arrows*). Magnification ×20. *Bar* 400 μm. **b** Lymphatics associated with the vomeronasal organ. Magnification ×100. *Bar* 100 μm. **c** Lymphatic plexus around the nasolacrimal duct. Magnification ×100. *Bar* 100 μm. **d** Lymphatics in the basal part of the nasal mucous membrane (*ns* nasal septum, *pa* palatine, *to* tongue). Magnification ×40. *Bar* 200 μm. **e**, **f** Immunofluorescence double-staining of lymphendothelial markers Lyve-1 (**e**, *green*) and Podoplanin (**f**, *red*) of regions near the nasolacrimal duct. Note the Lyve-1^+^/Podoplanin^+^ lymph vessels (*lv*) and Lyve-1^−^/Podoplanin^−^ blood vessels (*bv*). Magnification ×400. *Bar* 20 μm

**Fig. 4:**
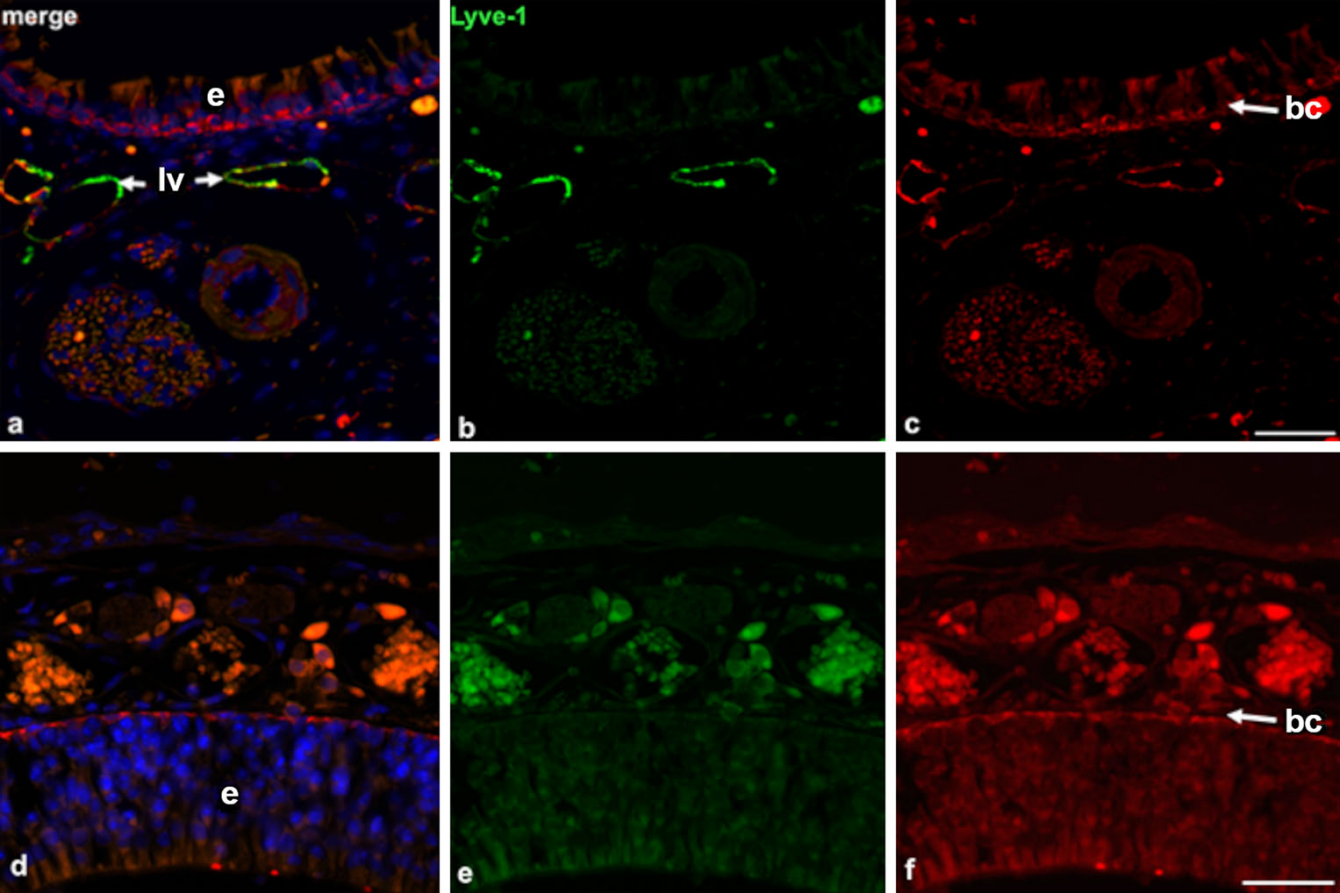
Lymphatics are absent in the upper parts of the nasal mucous membrane. **a–c** Immunofluorescence double-staining (*green* anti-Lyve-1, *red* anti-Podoplanin, *blue* 4,6-diamidino-2-phenylindole [Dapi]) of the lower part of the nasal mucous membrane. Note the epithelial layer (*e* in **a**, **d**) on top and Lyve-1^+^/Podoplanin^+^ lymph vessels (*lv*). Magnification ×400. *Bar* 20 μm. **d–f** Immunofluorescence double-staining (*green* anti-Lyve-1, *red* anti-Podoplanin, *blue* Dapi) of the upper part of the nasal mucous membrane. No double-positive lymph vessels can be detected. Note the Lyve-1^−^/Podoplanin^+^ basal cell layer (*bc* in **c**, **f**). Magnification ×400. *Bar* 20 μm

Much of the CSF is known to be drained via the *Fila olfactoria* into the nasal mucous membrane (Johnston et al. 2004). We were astonished to see that the nasal mucous membrane did not contain lymphatics, except for its most basal parts below the vomero-nasal organ (Figs. 3, 4). In contrast, the mucous membrane of the vomero-nasal organ and the inferior nasal turbinates presented with numerous lymphatics (Fig. 3b, d). Here, the nasolacrimal duct (NLD) discharges lacrimal fluid into the inferior nasal turbinate. All along its way to the medial angle of the eye, the NLD is covered by a dense network of lymphatics (Fig. 3c, e, f). This lymphatic network was found to be connected to the lymphatics of the eye (see below). The mucous membranes of the paranasal sinuses also contained lymphatics, as seen here for the maxillary and the sphenoidal sinus. The lymphatics of the latter were connected to those of the pharynx (data not shown).

**Fig. 5:**
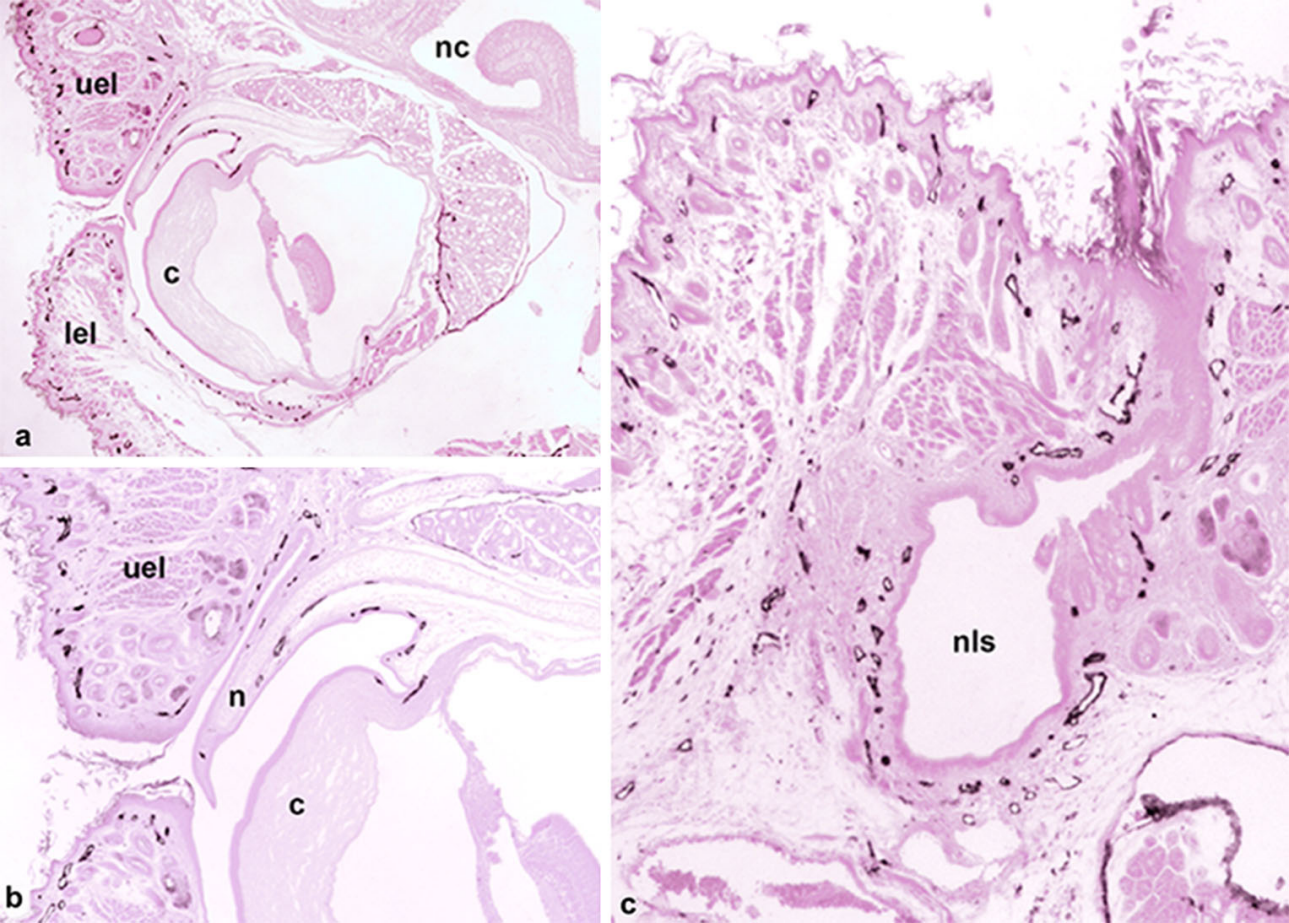
Lyve-1-positive lymphatics of the eye. **a** Overview showing the eye with the cornea (*c*), upper eyelid (*uel*) and lower eyelid (*lel*) and nasal cavity (*nc*). Magnification ×20. *Bar* 400 μm. **b** Higher magnification of **a**. Of note is the lymphatic network starting at the limbus of the cornea (*c*) and extending from the conjunctiva of the nicitating membrane (*n*) into the upper eyelid (*uel*). Magnification ×40. Bar 200 μm. **c** Medial angle of the eye and the nasolacrimal sac (*nls*), which discharges into the NLD and has a dense network of lymphatics. Magnification ×40. *Bar* 200 μm

In the eye, Schlemm’s canal has previously been identified as a lymphatic-like vessel (Aspelund et al. 2014; Kizhatil et al. 2014; D.-Y. Park et al. 2014; Ramos et al. 2007). Our staining shows that a Lyve-1-positive vessel is present in the limbus of the cornea and seems to be part of a lymphatic network of the conjunctiva, which also covers the murine nictitating membrane (Fig. 5a, b). Additionally, the eyelids contain a dense network of initial lymphatics. At the medial angle of the eye, these lymphatics form a continuum with the lymphatic networks accompanying the NLD towards the inferior nasal turbinate (Fig. 5c). Moreover, squamous epithelial cells, which appear to be homologous to the Tenon capsule of the human eye, are Lyve-1-positive.

**Fig. 6:**
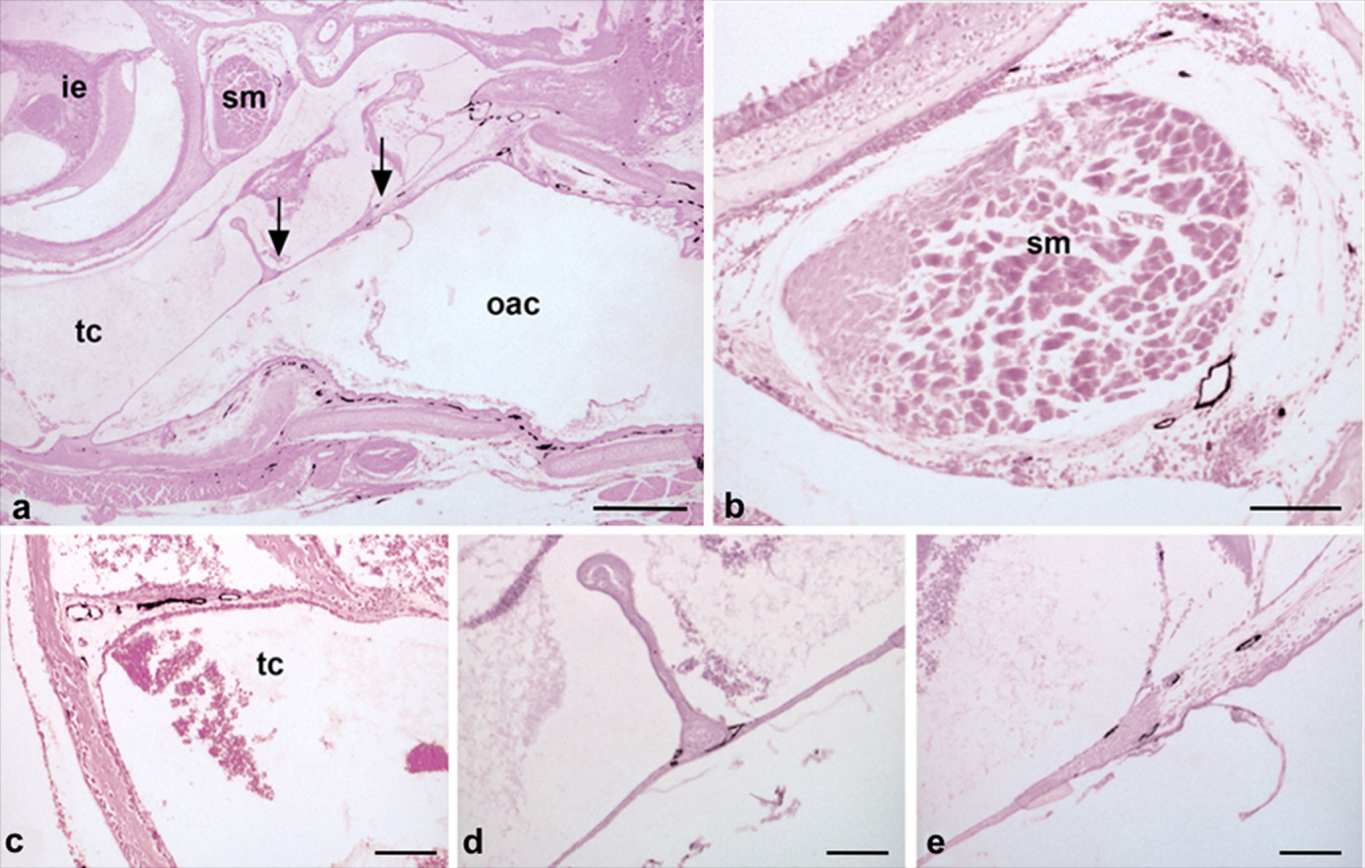
Lyve-1-positive lymphatics of the ear. **a** Overview showing the outer acoustic canal (*oac*), the tympanic cavity (*tc*), the inner ear (*ie*) and the stapedius muscle (*sm*). *Arrows* indicate tympanic membrane (shown at higher magnification in **d**, **e**). The outer acoustic canal (*oac*) contains a dense network of lymphatics. Magnification ×20. *Bar* 400 μm. **b** Stapedius muscle accompanied by lymphatic vessels. **c** Tympanic cavity (*tc*) drained by lymphatics. **d** Lymphatics are present at the contact site of the malleus and the tympanic membrane. **e** Lymphatics are present in the peripheral parts of the tympanic membrane. **c–e** Magnification ×100. *Bar* 100 μm

**Fig. 7:**
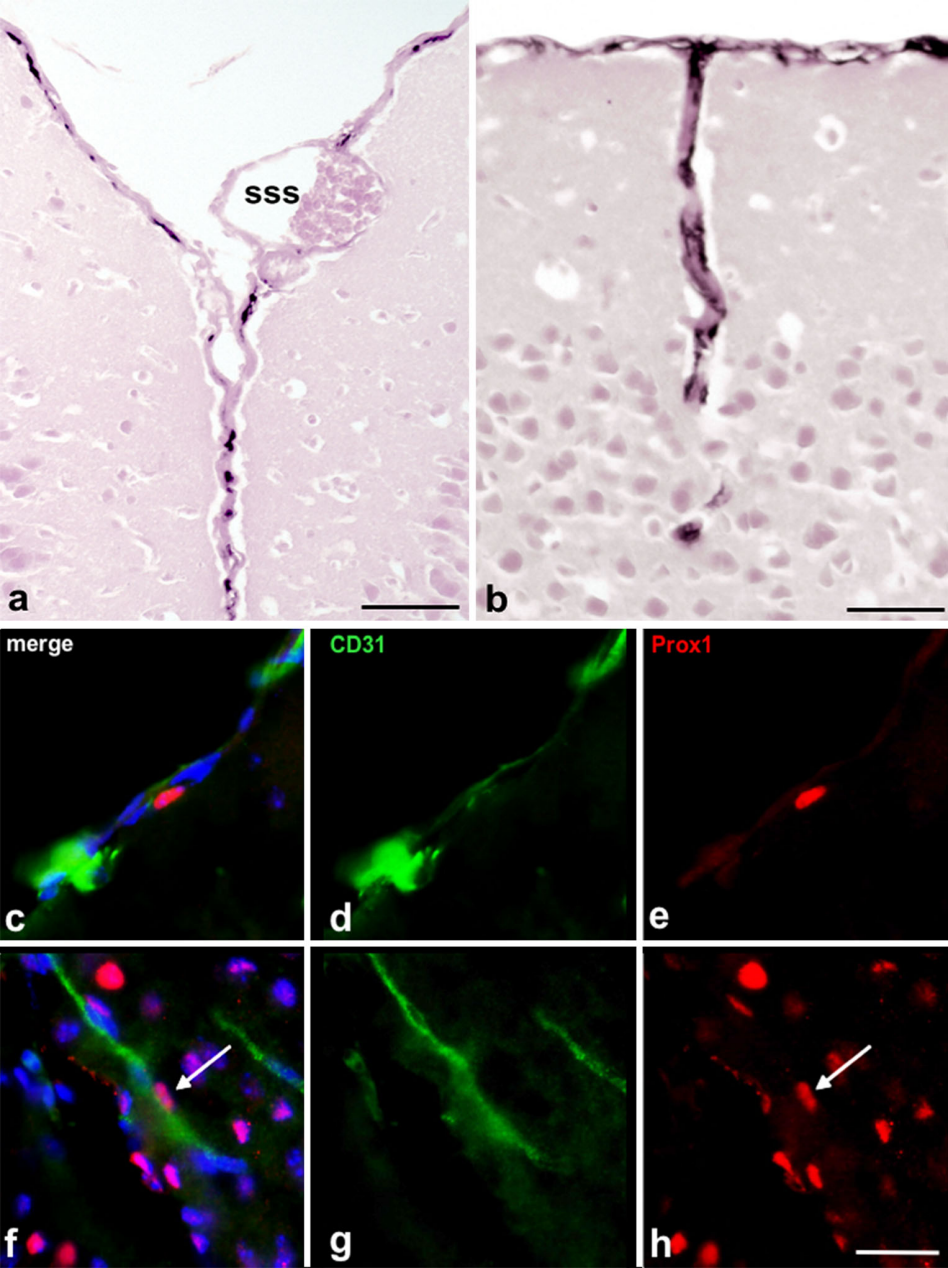
Lyve-1-positive lymphatics of the dura mater. **a** Telencephalic hemispheres and superior sagittal sinus (*sss*). Lyve-1-positive structures rarely show a distinct lumen. **b** Lyve-1-positive structures in deeper aspects of the telencephalic hemispheres. **a**, **b** Magnification ×200. *Bar* 50 μm. **c–e**, **f–h** Immunofluorescence double-staining (*green* anti-CD31, *red* anti-Prox-1, *blue* Dapi) of cryo-preserved mouse brains (day 14). **c–e** Example of Prox-1^+^ endothelial cell nucleus embedded in a CD31^+^ vessel located in the dura mater. **f–h** Example of Prox-1^+^ endothelial cell nucleus embedded in a CD31^+^ vessel located in deeper aspects of the telencephalic hemispheres. **c–h** Magnification ×400. *Bar* 20 μm

The lymphatics of the ear have rarely been described. In the outer auditory canal, a dense lymphatic network is present and proceeds into the peripheral parts of the tympanic membrane (Fig. 6a, e). Lymphatics are also present adjacent to the auditory ossicles (Fig. 6d), the stapedius muscle (Fig. 6b) and in the mucous membrane of the tympanic cavity (Fig. 6c). The lymphatics closest to the CNS are obviously those that are located in the dura mater, although a lumen is hardly visible in these vessels. Adjacent to the superior sagittal sinus, Lyve-1-positive structures are found in the central dura mater (Fig. 7a, c-e). Such structures can also be found at other sites, such as the dura mater surrounding the olfactory bulb (data not shown). Furthermore, Lyve-1-positive structures accompanying larger blood vessels entering the cortex of the brain can be detected (Fig. 7b, f-h).

## Discussion

We studied the pattern of the lymphovascular system in the head of 14-day-old mice by employing serial coronal sectioning of decalcified paraffin-embedded specimens and immunperoxidase staining with Lyve-1 and Podoplanin antibodies. The hyaluronan receptor Lyve-1 is a transmembrane glycoprotein that is highly expressed in initial lymphatics. Here, we could nicely differentiate between LECs, which were strongly positive and scattered ramified cells, which were weakly positive and most likely represented dendritic cells and macrophages (Ward et al. 2006). The second LEC marker used was Podoplanin, a surface glycoprotein named after its expression in kidney podocytes. As its expression is highly restricted to LECs (and not blood endothelial cells [BECs]), the co-expression of Podoplanin and Lyve-1 is a reliable diagnostic tool for characterization as lymphatic vessels (Box et al. 2002). However, we should mention here that we detected podoplanin expression in various other structures including basal cells of the epidermis, basal cells of the nasal mucous membrane, ducts of the salivary glands, ciliary bodies, epithelial cells of the lens, basal cells of cornea epithelium and endothelium, reticular cells of lymph nodes, perineurium and osteocytes.

To study the presence or absence of lymph vessels in the CNS, we additionally used double-staining of cryo-preserved mouse brains (day 14). Here, we used the markers CD31 (platelet endothelial cell adhesion molecule 1), which is an adhesion molecule that is enriched at the surface of BECs and LECs (Newman et al. 1990) and the homeobox-containing transcription factor Prox1, which is expressed in LECs but not in BECs (Wigle and Oliver 1999; Rodriguez-Niedenführ et al. 2001).

### Lymph nodes

We observed lymph nodes, other than the parotis, in the vicinity of the ear and between the parotis and submandibular gland at the floor of the mouth. Lymph nodes in mice have been known for decades. First descriptions were published in the early 1950s and 1960s (Barone et al. 1950; Cuq 1966; Kawashima et al. 1964); however, the nomenclature of lymph nodes is unfortunately not especially homogeneous. According to Van den Broeck et al. (2006), BALB/cAnCrl mice present mainly with four pairs of lymph nodes in the head and neck region. (1) The mandibular lymph nodes are located rostromedial to the sublingual and mandibular salivary glands, (2) the accessory mandibular lymph nodes occur dorsolateral to the mandibular lymph nodes, (3) the superficial parotid lymph nodes are found ventral to the external acoustic pore, cranioventral to the parotid salivary gland and dorsal to the junction between the superficial temporal and the maxillary vein and (4) the cranial deep cervical lymph nodes lie dorsal to the trachea between the sternocephalic, sternohyoid and digastric muscles (Van den Broeck et al. 2006). The afferent and efferent lymphatic vessels connected to these groups of lymph nodes have rarely been studied. Most probably, the accessory mandibular lymph nodes drain fluid from the lip, eyelid and tongue. The superficial parotid lymph nodes are supposed to drain the auricle, whereas the cranial deep cervical lymph nodes are supposed to drain deep regions of the head and the neck (Kawashima et al. 1964). The sinusoidal system in murine lymph nodes is known to be less complex than that in men. We hardly observed any trabecular sinuses, which also holds true for murine lymph nodes in other areas (Jang et al. 2013). A previous study, performed in 8-weeks-old nude mice, revealed that the lining endothelium of the subcapsular and medullary sinuses expresses the lymphatic endothelial markers Lyve-1 and Prox-1 (Liersch et al. 2012). In our study, the LECs of the marginal sinus were all found to be Podoplanin-positive but were heterogeneous as regards Lyve-1 expression. Those of the visceral lining were strongly Lyve-1-positive, whereas those of the parietal lining, adjacent to the capsule, were mostly Lyve-1-negative. Similarly, the human subcapsular and trabecular sinuses lack Lyve-1 expression (S.M. Park et al. 2014). In accordance with the findings in human lymph nodes, HEVs are Lyve-1 positive (Wrobel et al. 2005).

### Dental pulp

In the mouse, the oral and pharyngeal mucous membranes contain a dense lymphatic vascular network. However, the presence of lymphatics in the dental pulp is controversial. Some groups claim that a lymphatic drainage system exists in the dental pulp of diverse species. Various immunohistochemical methods, immersion of teeth in Patent Blue and ultrastructural analyses by electron microscopic techniques have been used to verify this hypothesis (Bernick 1977; Bishop and Malhotra 1990; Marchetti et al. 1991; Matsumoto et al. 2002; Oehmke et al. 2003). Nevertheless, more recent studies in this field utilizing immunohistochemical staining with the common LEC markers Lyve-1, Prox1, VEGFR-3, or D2-40 have been unable to detect lymphatic structures in the human or dog dental pulp (Gerli et al. 2010; Martin et al. 2010). Here, we show that the dental pulp of mice does not contain initial lymphatics. However, interstitial fluid is obviously drained into lymphatics that accompany dental arteries and nerves, as shown here for the inferior alveolar nerve and artery.

### Ear

The lymphatic system of the ear has not been very well described to date. The middle ear has previously been shown to contain lymphatics that drain to the cervical lymph nodes (Lim and Hussl 1975). In contrast, the lymphatic drainage of the perilymph is largely unidentified. Tracer solution injected into the cerebellomedullary cistern of guinea pigs is detectable in the cochlear perilymph and later in the cervical lymph nodes (Arnold et al. 1972). Together with the electron microscopic finding that perilymph has contact with the lymphatics of the middle ear mucosa, the preferred theory was originally that the lymph fluid of the inner ear is drained to the middle ear and its lymphatic vessels (Arnold and Ilberg 1972). Later, a direct connection of the inner ear to the cervical lymph nodes was suggested (Yimtae et al. 2001). We detected a dense network of lymphatics in the outer auditory canal and lymphatic vessels in the peripheral parts of the tympanic membrane, near the auditory ossicle and the stapedius muscle. However, we were not able to judge whether the lymph of the inner ear is directly drained to the adjacent lymph nodes.

### Drainage of CSF

The problem of the way in which lymphatics of the head contribute to the clearance of the cerebrospinal fluid (CSF) is currently highly debated. The common view used to be that CSF, after being formed in the choroid plexus, flows through the ventricles and the subarachnoid space to its final site of reabsorption in the blood stream. This absorption was thought to occur mainly through the arachnoid villi of the dural sinuses, along cranial nerve sheaths, or through the nasal lymphatics (Abbott 2004; Praetorius 2007; Koh et al. 2005). Brain interstitial solutes have been suggested to be cleared to the CSF by a bulk flow of interstitial fluid, diffusely through the brain tissue (Abbott 2004; Cserr et al. 1981; Syková and Nicholson 2008). However, these theories have recently been modified by experts in the field, as the lack of a pathway for interstitial solute clearance in the brain, as is present in other tissues, would be surprising. In particular, the brain has a high metabolic rate and needs the rapid clearance of interstitial fluid, because of the sensitivity of neurons and glial cells to alterations in their extracellular environment. Increasing evidence has raised the possibility that the lymphatic system has an important function for the clearance of brain extracellular solutes but the explicit way in which solutes from the brain interstitium pass to the CSF is still not clear. Several groups have performed studies in which they injected tracers into the CSF or the brain parenchyma of various species. One major statement arising from most of these studies is that the tracers are drained to different lymph nodes in the head and neck region and that the cibriform plate is a central structure of this clearance pathway (Cserr and Knopf 1992; Kida et al. 1993). Some studies have shown that CSF is drained along the *Fila olfactoria* into the nasal mucous membrane and since the deep cervical lymph nodes were found to contain tracers shortly after injection, a direct connection of the CSF and the mucosal lymphatics has been proposed (Johnston et al. 2004; Zakharov et al. 2003, 2004; Koh et al. 2006). Interestingly, we could show, in our study, that lymphatics do not exist in most parts of the nasal mucous membrane, except for the most basal parts of the inferior turbinates. Conflicting results might be caused by technical limitations of the performed methods. The injection of Evan’s Blue, for example, faces the problem that the dye tends to dissociate from the coupled protein and that it cannot be prepared for histology. The injection of Microfil, another well-known tracer for lymphatic vessels, must be injected *post mortem* with pressure. This procedure often leads to the disruption of the fragile lymphatic networks. Therefore, neither method allows the precise identification of lymphatic vessels.

**Fig. 8:**
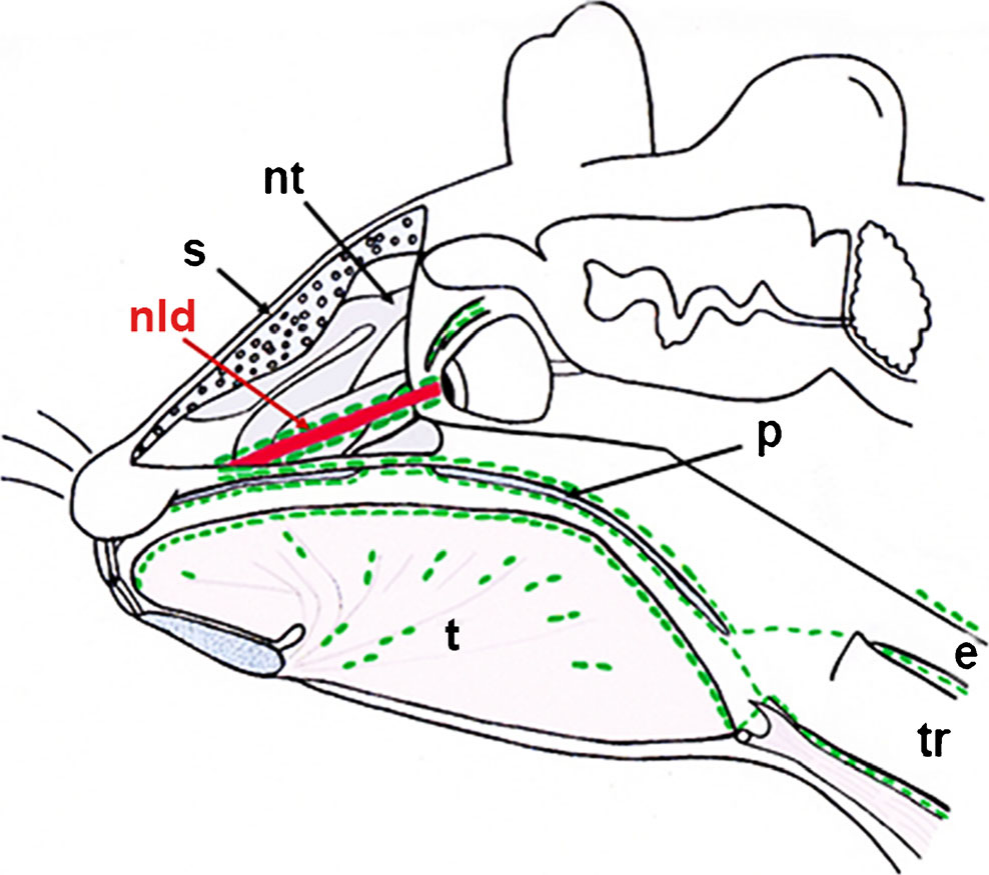
Overview of main lymph drainage pathways in the mouse head. Representation of the newly described drainage pathway along the nasolacrimal duct along the palatine into a supposed lymph plexus in the area at the beginning of the esophagus and trachea. Routes of lymph vessels are indicated in *green*, whereas the nasolacrimal duct is shown in *red* (*s* septum, *nt* nasal turbinates, *nld* nasolacrimal duct, *t* tongue, *p* palatine, *e* esophagus, *tr* trachea)

An interesting outcome of our study is the identification of a dense lymphatic network that accompanies the NLD and that lies in direct contact with the lymphatics of the inferior nasal turbinates. This drainage pathway represents a direct connection of the dense lymphatic networks located in the conjunctiva and the dermis of the eyelid with the dense lymphatic network of the palatine/oral mucous membrane, which further drains into superficial and deep cervical lymph nodes. Because of the embryonic development of the NLD, we propose that the lymphatics along the NLD represent a continuation of the dermal lymphatic network (Moore et al. 2015). The NLD develops at the contact site of the frontonasal and maxillary prominences. Its epithelium is a derivative of the epidermis and its lamia propria is a continuation of the dermis; based on our findings, its lymphatics appear to be a continuation of the dermal lymphatic network. This result supports the view of the drainage of CSF via the arachnoid space along the optic nerve to the eyeballs (Cserr and Knopf 1992; Kida et al. 1993; Lüdemann et al. 2005). Taking into consideration the missing lymphatic network in the nasal mucous membrane and the newly described pathway along the NLD, we hypothesize that the upper parts of the nasal mucous membrane do not represent the main pathway for CSF to the lymph nodes of the head and neck region. We can imagine that, instead, the bulk of CSF that drains along the olfactory nerves is used for the moisturization of the respiratory air, whereas the CSF that is drained to the cervical lymph nodes flows along with the NLD (Fig. 8). One hint for the functionality of the lymphatic drainage pathway along the NLD can be seen as carcinomas of the efferent tear lanes are known to metastasize into surrounding structures including the eye socket and the eye lid and into the nasal cavity and the paranasal sinuses (Heindl et al. 2010a; Parmar and Rose 2003; Pe’er et al. 1994; Stefanyszyn et al. 1994). Moreover, the metastasis of other ocular tumors to the lymph nodes of the head and neck regions might be explained by this drainage pathway, e.g., squamous cell carcinoma of the conjunctiva (Tabbara et al. 1988; Heindl et al. 2010b), intraocular melanoma (Dithmar et al. 2000), or conjunctival melanoma (Heindl et al. 2011).

In addition to the CSF drainage pathway through the head, the presence or absence of lymphatics in the CNS has currently drawn new interest. Although previous publications have mentioned dural lymphatics in the past (Kida et al. 1993; Iliff et al. 2012), recent studies describing a dural lymphatic system have brought this topic into the focus of debate once again. Claiming the presence of the direct drainage of brain interstitial fluid into lymphatic vessels inside the meninges, Aspelund et al. and Louveau et al. question the so-far accepted lack of lymphatic vasculature in the CNS (Aspelund et al. 2015; Louveau et al. 2015). In our study, we have been able to confirm the existence of such a dural lymphatic system by Lyve-1 staining and Prox-1/CD31 double-staining of cryo-preserved mouse brains. Nevertheless, the dural lymphatic network might not represent the only lymphatic vasculature in the CNS. A two-photon microscopic study by Iliff et al. (2012) showed that fluorescent tracers injected into the cisterna magna of anesthetized mice enter the brain along the pial surface and along paravascular spaces that surround arteries penetrating the brain but do not enter the surrounding interstitial spaces. In another experimental setup, the same group revealed that brain interstitial fluid can be cleared along paravenous drainage pathways, with astrocytes and, especially, the water channel aquaporin-4 in astrocytic endfeet playing a substantial role in the clearance process (Iliff et al. 2012). In our study, we also obtained initial hints for the presence of lymphatics in the meningeal septae penetrating into the brain. The morphology and functionality of these lymphatics need to be reviewed but if confirmed, this could offer new approaches for studying the etiology of neuroinflammatory and neurodegenerative diseases.

### Concluding remarks

Our study shows that staining of serial paraffin sections with LEC-specific antibodies can successfully be performed in 14-day-old mice after decalcification of the tissue. We describe a new lymphatic vascular system that lies along the NLD and that connects the lymphatics of the eye with those of the inferior nasal and the palatinal regions.
